# Conservation of MAP kinase activity and MSP genes in parthenogenetic nematodes

**DOI:** 10.1186/1471-213X-10-51

**Published:** 2010-05-17

**Authors:** Peter Heger, Michael Kroiher, Nsah Ndifon, Einhard Schierenberg

**Affiliations:** 1 Zoological Institute, University of Cologne, Zülpicher Strasse 47b, 50674 Köln, Germany

## Abstract

**Background:**

MAP (mitogen-activated protein) kinase activation is a prerequisite for oocyte maturation, ovulation and fertilisation in many animals. In the hermaphroditic nematode *Caenorhabditis elegans*, an MSP (major sperm protein) dependent pathway is utilised for MAP kinase activation and successive oocyte maturation with extracellular MSP released from sperm acting as activator. How oocyte-to-embryo transition is triggered in parthenogenetic nematode species that lack sperm, is not known.

**Results:**

We investigated two key elements of oocyte-to-embryo transition, MSP expression and MAP kinase signaling, in two parthenogenetic nematodes and their close hermaphroditic relatives. While activated MAP kinase is present in all analysed nematodes irrespective of the reproductive mode, MSP expression differs. In contrast to hermaphroditic or bisexual species, we do not find MSP expression at the protein level in parthenogenetic nematodes. However, genomic sequence analysis indicates that functional MSP genes are present in several parthenogenetic species.

**Conclusions:**

We present three alternative interpretations to explain our findings. (1) MSP has lost its function as a trigger of MAP kinase activation and is not expressed in parthenogenetic nematodes. Activation of the MAP kinase pathway is achieved by another, unknown mechanism. Functional MSP genes are required for occasionally emerging males found in some parthenogenetic species. (2) Because of long-term disadvantages, parthenogenesis is of recent origin. MSP genes remained intact during this short intervall although they are useless. As in the first scenario, an unknown mechanism is responsible for MAP kinase activation. (3) The molecular machinery regulating oocyte-to-embryo transition in parthenogenetic nematodes is conserved with respect to *C. elegans*, thus requiring intact MSP genes. However, MSP expression has been shifted to non-sperm cells and is reduced below the detection limits, but is still sufficient to trigger MAP kinase activation and embryogenesis.

## Background

Throughout the animal kingdom, female gametes interrupt their development during oogenesis at various stages of meiosis. In response to external stimuli, this arrest is released, and oocyte maturation can take place. Then oocytes resume meiotic divisions, ovulate and get competent for fertilisation.

An important step during oocyte maturation of all animals is MAP kinase activation (reviewed in [1-3]). MAP kinases are ubiquitous serine-threonine protein kinases expressed in all eukaryotic cells [4,5] and can be divided into five groups: The Erk1/2, p38, Jnk, Erk3/4, and Erk5 subfamilies [5]. They are activated by MAP kinase kinase-mediated dual phosphorylation on two distinct amino acids, Threonine and Tyrosine, in a T-X-Y motif of the activation loop [6]. Phosphorylation induces a rotation between the N- and C-terminal domains that activates the kinase [7]. Activation is accompanied by a partial translocation to the nucleus [8,9] for phosphorylation of nuclear targets which mainly consist of transcription factors [5]. MAP kinase inactivation on the other hand is accomplished by MAP kinase phosphatases that specifically recognise and remove the dual phosphorylation [10].

There are MAP kinase orthologs from several subfamilies present in *C. elegans*. The Erk1/2 ortholog MPK-1 [11,12] is the best studied representative and functions with its upstream cascade members LET-60 Ras (Ras-related GTPase), LIN-45 Raf (MAP kinase kinase kinase) and MEK-2 (MAP kinase kinase) in vulval cell fate specification, cell migration, and oocyte-to-embryo transition (reviewed in [13,14]).

MSP is a small basic protein of ~15 kDa (for review see [15]). It was first described as a major component of *C. elegans *sperm representing 15% of its total protein content [16]. In *C. elegans*, MSP comprises a large multigene family of about 50 highly conserved members [17] including more than 20 pseudogenes. The number of MSP genes detected in other nematodes is variable, from one in *Ascaris suum *to 1-13 in other mammalian intestinal parasites, 1-4 in filarial nematodes or 5-12 in plant and insect parasitic species [18,19]. MSP sequences are highly conserved in all nematodes [15]. All MSP genes of *C. elegans *are expressed at the same time and only during the terminal stages of spermatogenesis [20,21]. Restriction of MSP expression to male animals or their spermatocytes is also known for *Oesophagostomum dentatum*, *Brugia malayi*, *Dictyocaulus viviparus *and *Ascaris suum *[19,22-24].

Besides its functions in sperm structure and motility [25-27], Greenstein and colleagues have shown that MSP has a range of extracellular signaling properties [28]. In oocytes, MSP signaling induces via the receptor tyrosine kinase VAB-1 the activation of MAP kinase and subsequently meiotic maturation and cell cycle progression [28,29]. It promotes muscle contractions in somatic gonadal sheath cells to facilitate ovulation [28]. In parallel, MSP signaling antagonises inhibitory signals from both, gonadal sheath cells and oocytes, that prevent meiotic maturation in the absence of sperm [29,30]. Gonadal sheath cells accordingly seem to function as the major initial sensor of MSP and control all MSP-dependent meiotic maturation events, potentially via multiple classes of G-protein-coupled receptors and communication through gap junctions [31].

These reports from the *C. elegans *literature highlight that resumption of meiosis and ovulation depend on sperm and sperm-released factors, thereby avoiding cost if no sperm is available. However, within the phylum Nematoda a variety of reproductive strategies exists. There is general consent that sexual reproduction is the ancestral and most widespread condition. In contrast, parthenogenetic reproduction and hermaphroditism are derived modes that have evolved within the phylum Nematoda several times independently [32,33].

Whether or not the molecules regulating the *C. elegans *oocyte-to-embryo transition are functionally conserved in nematodes with different reproductive modes, is not known. Using *C. elegans *as a reference, we therefore wanted to investigate two key steps of oocyte-to-embryo transition, MAP kinase activation and MSP signaling, in parthenogenetic nematodes where sperm is absent.

## Results

### MAP kinase genes are conserved in parthenogenetic nematodes

**Figure 1:**
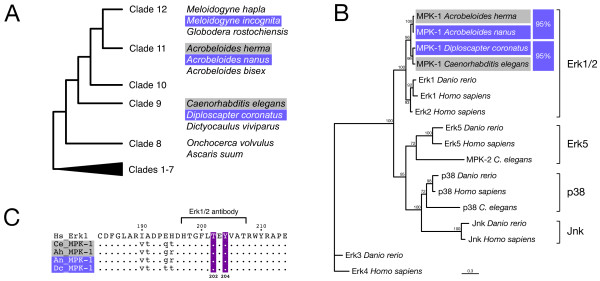
**MAP kinase sequence is conserved in parthenogenetic nematodes**. **A: **Phylogenetic tree of the phylum Nematoda. Species mentioned in this study are indicated and mapped to their respective clade. Clades 1-7 are collapsed into one branch. Colour assigns parthenogenetic (violet) or hermaphroditic reproduction (grey) through all figures. **B: **Phylogenetic analysis of MAP kinase genes from hermaphroditic (*C. elegans *and *Acrobeloides (herma)*) and parthenogenetic (*A. nanus *and *D. coronatus*) nematodes using maximum likelihood (model: RtREV). Significances at branches are indicated. All newly isolated nematode MAP kinase genes belong, like *C. elegans *MPK-1, to the Erk1/2 subfamily of MAP kinases. They group in line with the inferred phylogenetic position, not according to the underlying reproductive mode. *C. elegans *MPK-2, to which a function has not been ascribed so far, clusters to the Erk5 subfamily. **C: **Sequence alignment of the activation loop of human and nematode Erk1/2 MAP kinase proteins. Amino acids T202 and Y204 (numbering after human Erk1), which get phosphorylated during activation, are indicated (red). The epitope recognised by the antibody against active MAP kinase, including the dual phosphorylation motif, is highly specific for Erk1/2 family members and perfectly conserved in nematode MAP kinases irrespective of the reproductive mode.

As MAP kinase activation is mediated by sperm in *C. elegans *[28,34], we reasoned that parthenogenetic nematodes that lack sperm might differ from hermaphrodites like *C. elegans *with respect to MAP kinase properties. To test this assumption, we cloned MAP kinase genes of the parthenogenetic nematode *Acrobeloides nanus *and of its close relative with hermaphroditic reproduction, *Acrobeloides (herma)*, as well as the MAP kinase gene of a second parthenogenetic nematode, *Diploscapter coronatus*, a close relative of *C. elegans *(see Figure 1A; phylogeny after [35]). As only Erk1/2 MAP kinases are involved in oocyte maturation in *C. elegans *[34] and other animals [36-40], we wanted to ensure that the cloned MAP kinase sequences belong to this subfamily. Thus, we performed a phylogenetic analysis with a sequence set representing the five MAP kinase subfamilies. The resulting phylogenetic tree confirmed the placement of the newly isolated MAP kinase sequences to the Erk1/2 subfamily (Figure 1B). It further suggests that *C. elegans *MPK-2, whose function is not known so far [41], is a member of the Erk5 subfamily of MAP kinases. This is the least characterised mammalian MAP kinase pathway and known to be activated by oxidative stress and hyperosmolarity [42]. According to our phylogenetic tree, MPK-1 is the only Erk1/2 MAP kinase ortholog present in *C. elegans *suggesting that our cloned MAP kinase sequences, too, represent Erk1/2 orthologs with a possible role in oocyte maturation. Sequence comparison revealed that the MAP kinase sequences between phylogenetically related, but reproductively different pairs *A. nanus*/*Acrobeloides (herma) *and *C. elegans*/*D. coronatus *are slightly more similar to each other than sequence pairs derived from nematodes with identical reproduction (95% vs. 92%, Figure 1A, B). The amino acid sequence of the entire activation loop is highly conserved in the MAP kinases of all nematode species considered here (Figure 1C). In particular, T202 and Y204, which are phosphorylated during activation, are present in all MAP kinases and match the T-X-Y dual phosphorylation motif found in *C. elegans *MPK-1 and other Erk1/2 family members (Figure 1C). Thus, the considerable sequence conservation between hermaphroditic and parthenogenetic MAP kinases rejects the possibility of a specific alteration in parthenogenetic nematodes.

### MAP kinase activation is conserved in parthenogenetic nematodes

**Figure 2:**
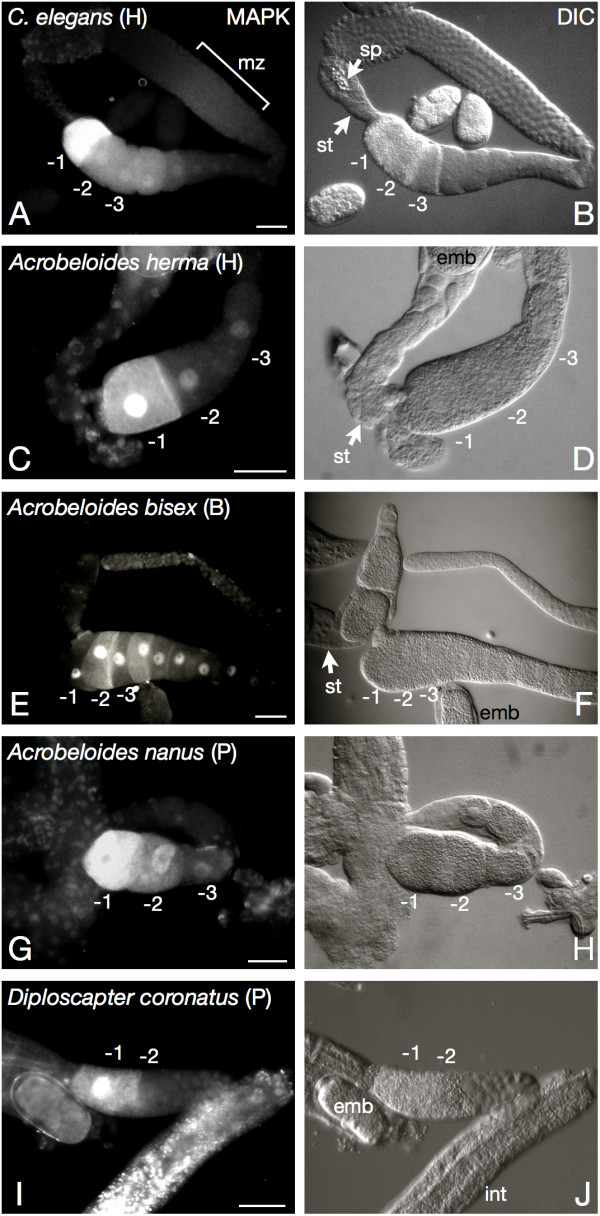
**MAPK activation is conserved in parthenogenetic nematodes**. Gonad preparations of parthenogenetic (P), hermaphroditic (H) and bisexual (B) nematode species stained for active MAP kinase (left panel; corresponding DIC picture: right panel). In all five nematode species with three different modes of reproduction, MAP kinase activation can be detected in proximal oocytes as described for the *C. elegans *system [28]. It is therefore independent of the reproductive strategy. In *C. elegans*, MAP kinase activation is biphasic and can also be observed in the meiotic zone (mz) of the syncytial gonad (panel A, bracket; [28,47]). The pronounced nuclear staining in *Acrobeloides *species might reflect early MAP kinase dependent gene expression. sp: sperm. st: spermatheca. int: intestine. emb: embryo. -1, -2, -3: proximal oocytes, -1 is most proximal. All gonads are oriented with proximal to the left. Bar: 20 *μ*m.

Since the MAP kinase activation loop including the dual phosphorylation motif is conserved in parthenogenetic nematodes (Figure 1C), we wanted to know whether MAP kinase activation is detectable in these nematodes despite the lack of sperm. Therefore, we stained gonad preparations of our nematode set with a monoclonal antibody highly specific for the activation loop of diphosphorylated Erk1/2 MAP kinase. Our experiments revealed that MAP kinase is active in late-stage oocytes of all species regardless of the reproductive mode (Figure 2). MAP kinase stainings of *C. elegans *gonads confirmed already published data [28,29,34] and gave a positive signal in proximal oocytes and the gonadal loop region (Figure 2A). This indicates that our experimental setup gives the anticipated results. As previously reported [34], we observed variable patterns of activated MAP kinase in the proximal oocytes of *C. elegans *(not shown), but also in the three *Acrobeloides *species. MAP kinase activation sometimes extended over three or four proximal oocytes (-1 to -4), occasionally interrupted by a non-activated cell, or could be restricted to the -2 or -3 oocyte without a signal in -1. We noticed such staining patterns in 4/11 (*A. nanus*) and 11/22 cases (*Acrobeloides (herma)*). As an example, an unusual staining of the second and fourth, but not the first, oocyte from the *Acrobeloides (bisex) *gonad is shown (Figure 2E). It likely represents a snapshot shortly after activation of the -1 oocyte has been completed. In contrast to other nematodes, exclusively the most proximal oocyte was stained in *D. coronatus *(13/13 cases; Figure 2I). This difference may be attributed to the shorter gonadal arms of *D. coronatus *where just a single or at most two large, yolk containing oocytes are present (Figure 2I, J) compared to a row of 5-10 in the other species.

Furthermore, the percentage of stained oocytes was considerably lower in *D. coronatus *than in *C. elegans *or in the *Acrobeloides *species (roughly 10% vs. 50%). While *C. elegans *oocytes mature at a fast rate of one every 23 min [43], this interval is several times longer in *D. coronatus *(data not shown) giving a likely explanation for the less frequent staining of oocytes in this species.

We sometimes observed in the *Acrobeloides *species a more pronounced MAP kinase signal in the nuclei of late-stage oocytes than in *C. elegans *(Figure 2C, E). This might be a consequence of the need for early transcription in *Acrobeloides *embryos [44] as compared to *C. elegans *[45].

MAP kinase activation is also required at an earlier stage in developing *C. elegans *oocytes, for the exit from pachytene of the first meiotic prophase [46], after which it is rapidly turned off. Therefore, a characteristic biphasic pattern of MAP kinase activation can be observed in the *C. elegans *germline (Figure 2A; [28,47]). Like *C. elegans *and other non-parthenogenetic nematodes the parthenogenetic species *A. nanus *and *D. coronatus *undergo meiosis leading to the formation of polar bodies [48]. We wondered whether an analogous early activation of MAP kinase in conjunction with meiosis is detectable in the distal gonads of our parthenogenetic nematodes. Our preliminary data do not support the presence of a biphasic activation (not shown) suggesting that oocyte development as well as meiotic cell cycle regulation may be different in parthenogenetic vs. non-parthenogenetic nematodes.

In conclusion, activation of the Erk1/2 MAP kinase pathway is a hallmark of oocyte maturation in parthenogenetic nematodes as it is in many other animals. Therefore, MAP kinase activation in proximal oocytes does not mirror differences in the reproductive mode.

### Major sperm protein is not detectable in parthenogenetic nematodes

**Figure 3:**
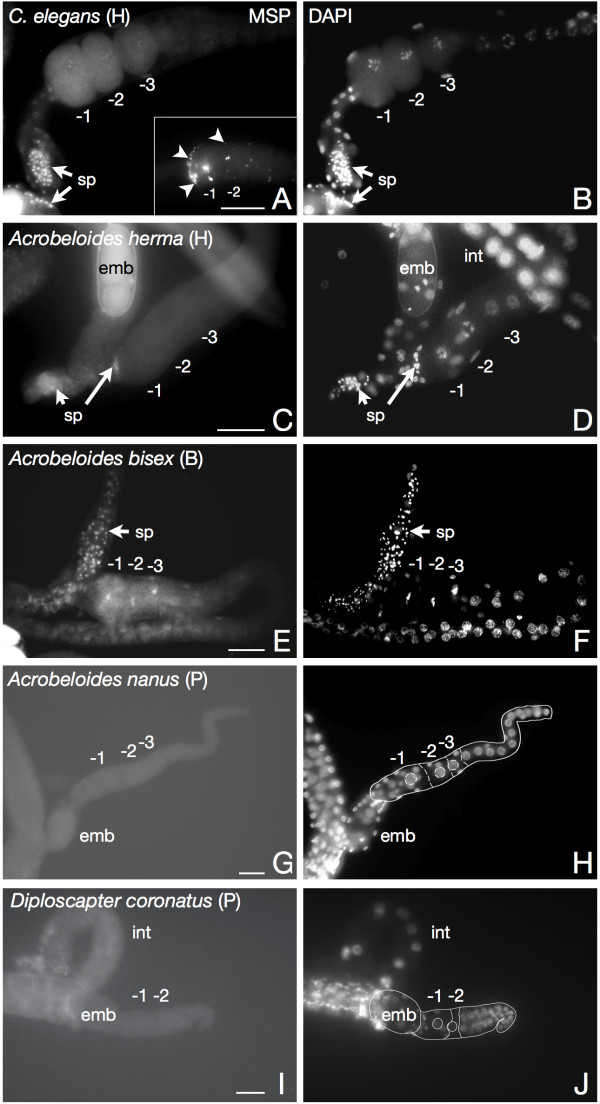
**Immunofluorescence analysis of MSP in nematode gonads**. Dissected gonad preparations of parthenogenetic (P), hermaphroditic (H) and bisexual (B) nematodes were doubly stained for MSP (left panel) and DNA (DAPI, right panel). While sperm associated MSP is revealed in the hermaphrodites *C. elegans *(panel A) and the distantly related species *Acrobeloides (herma) *and *Acrobeloides (bisex) *(panels C, E; arrows), a signal cannot be spotted in the parthenogenetic species *A. nanus *and *D. coronatus *(panels G, I). Arrowheads in panel A, inset, indicate puncta at the surface of the -1 oocyte resembling extracellular MSP as published previously [49]. The inset of panel A and panels G-J are from the same experiment, panels A-F from another. The photographs of parthenogenetic animals were overexposed to demonstrate the lack of staining, and their gonadal shape is outlined for better orientation. sp: sperm. st: spermatheca. int: intestine. emb: embryo. -1, -2, -3: proximal oocytes, -1 is most proximal. All gonads are oriented with proximal to the left. Bar: 20 *μ*m.

**Figure 4:**
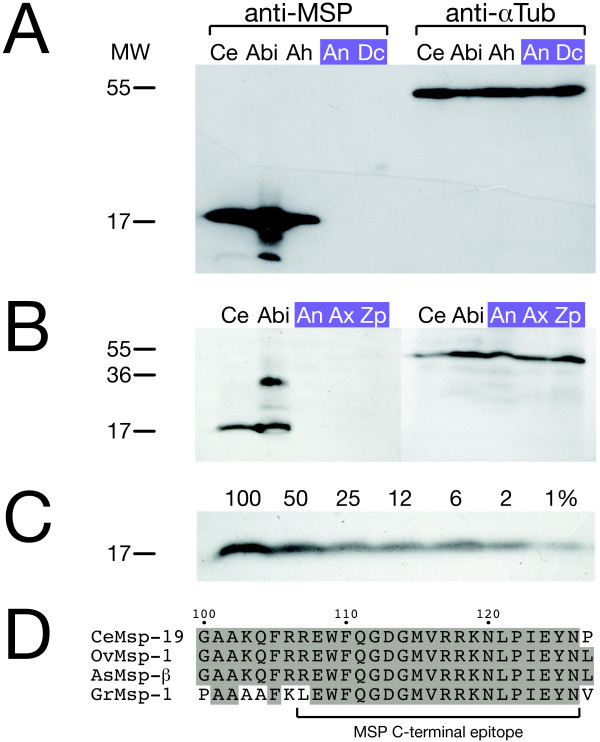
**MSP analysis in whole nematode extracts**. **A: **MSP is not detectable by Western blot in lysates of the parthenogenetic nematodes *A. nanus *and *D. coronatus*. Lysates of nematodes with various reproductive modes were, in roughly equal amounts, subjected to SDS-PAGE and Western blotting. One membrane of a duplicate set was incubated with monoclonal anti-MSP antibody, the other as a control with anti-*α*-tubulin antibody. While MSP is recognised in *C. elegans *and hermaphroditic or bisexual representatives of the distant *Acrobeloides *genus, none of the parthenogenetic species (violet) displays a signal. Ce: *C. elegans*; Abi: *Acrobeloides (bisex)*; Ah: *Acrobeloides (herma)*; An: *A. nanus*; Dc: *D. coronatus*. MW: molecular weight [kDa]. **B: **MSP is not detectable in additional parthenogenetic nematode species. As above, roughly equal amounts of nematode lysate were subjected to SDS-PAGE and Western blotting. While MSP is recognised in *C. elegans *and the sperm producing *Acrobeloides (bisex)*, two further parthenogenetic species (violet) do not reveal a signal. Ce: *C. elegans*; Abi: *Acrobeloides (bisex)*; An: *A. nanus*; Ax: *A. maximus*; Zp: *Z. punctata*. MW: molecular weight [kDa]. **C: **MSP is detectable over a wide range of concentrations. Serial dilutions of *C. elegans *lysates were subjected to SDS-PAGE and Western blotting using the monoclonal anti-MSP antibody. Numbers above the lanes indicate the relative amount of lysate used. 100% approximately correspond to 8 mg of worms, the amount used in experiments 4A and 4B. **D: **The C-terminal epitope recognised by the MSP antibody is widely conserved throughout the Nematode phylum. An alignment of *C. elegans *and other MSP sequences, derived from nematodes of clades 8-12 (Figure 1A), demonstrates the almost perfect conservation of MSP C-termini in a wide phylogenetic range. Accession numbers: Ce (*C. elegans*, AAC26926.1); Ov (*Onchocerca volvulus*, J04662.1); As (*Ascaris suum*, P27440.3); Gr (*Globodera rostochiensis*, AAA29146.1).

MAP kinase sequences and MAP kinase activation in the germline of parthenogenetic species appear similar to the model system *C. elegans*. Consequently, we asked whether MSP, the trigger of this activation in *C. elegans *[28], is also present in parthenogenetic nematodes. To test this possibility, we performed immunofluorescence analysis of nematode gonads (Figure 3) with a monoclonal antibody against the highly conserved C-terminus of MSP (Figure 4D; [49]). Staining of the hermaphroditic *C. elegans *gonad gives the expected signal of sperm-associated MSP coinciding with the DAPI signal of sperm nuclei (Figure 3A, B). The same could be observed after staining the gonads of hermaphroditic and bisexual *Acrobeloides *species (Figure 3C, D and 3E, F) indicating that the *C. elegans *antibody is able to recognise MSP from distant groups. In the cases where we detected sperm nuclei (Figure 3B, D, F), the corresponding MSP signal appeared slightly blurred. This is consistent with the demonstration of cytoplasmatic and extracellular MSP in *C. elegans *[49] and suggests that the extracellular signaling function of MSP might be conserved in other nematode groups. In addition to the sperm-associated signal, the MSP antibody marked extracellular puncta at the surface of the -1 and occasionally the -2 oocyte (Figure 3A, inset). A similar localisation of secreted MSP in the *C. elegans *gonad has been reported in a previous paper [49] indicating an adequate sensitivity of our experimental setup. In contrast to the sexual species, we never observed such puncta or other MSP-related signals in gonad preparations of the parthenogenetic nematodes *A. nanus *and *D. coronatus *(Figure 3G, I). Likewise, we could not detect sperm nuclei in these species (Figure 3H, J; [48]). As spermatocytes are the only known cells in nematodes where MSP is expressed [20-22,24], the absence of MSP is expected in nematodes lacking sperm.

To confirm the absence of MSP protein with an alternative method, we performed Western blot analysis of worm lysates using the C-terminal MSP antibody. In agreement with our immunostaining results, we could neither detect MSP signals in the two parthenogenetic species *A. nanus *and *D. coronatus *(Figure 4A) nor in two additional parthenogenetic species, *Acrobeloides maximus *and *Zeldia punctata *(Figure 4B), even if worm lysate was used in large excess (not shown). In contrast, a clear MSP signal at the size of the *C. elegans *control reveals the presence of MSP in the sperm-containing species *Acrobeloides (bisex) *and *Acrobeloides (herma)*. This demonstrates cross-reactivity of the antibody to MSPs from other species as expected from the conservation of MSP C-termini in a wide range of nematodes (Figure 4D). To rule out insufficient sensitivity of our MSP detection assay, we performed a Western blot with serial dilutions of *C. elegans *lysate. This experiment illustrates that the antibody still detects MSP if the starting lysates are diluted hundredfold (Figure 4C).

Additional smaller bands in the *Acrobeloides (bisex) *lane (Figure 4A) likely represent cleavage products of MSP which have also been reported for *C. elegans *[49]. In some experiments, we observed an additional larger signal at 35 kDa. As MSP is known to form extremely stable dimers [50], this signal likely indicates dimerized MSP (Figure 4B, *Acrobeloides (bisex) *lane).

In conclusion, our results show that MSP is not detectable and thus appears to be absent at the protein level in the parthenogenetic species we analysed.

### Intact MSP genes are present in parthenogenetic nematodes

Parthenogenetic nematodes do not undergo spermatogenesis during which MSP is normally expressed. Indeed, our experiments gave no evidence for its expression. We therefore expected that they do not require MSP, allowing the accumulation of mutations in MSP genes over time. A decay of genes specific to sex and recombination is predicted as an effect of the loss of sex on the eukaryotic genome [51]. To investigate this hypothesis, we cloned MSP genes from the parthenogenetic nematode *A. nanus*, from its hermaphroditic sister species *Acrobeloides (herma) *and from the parthenogenetic *D. coronatus*. We isolated and characterised several MSP genes from all three nematodes.

**Figure 5:**
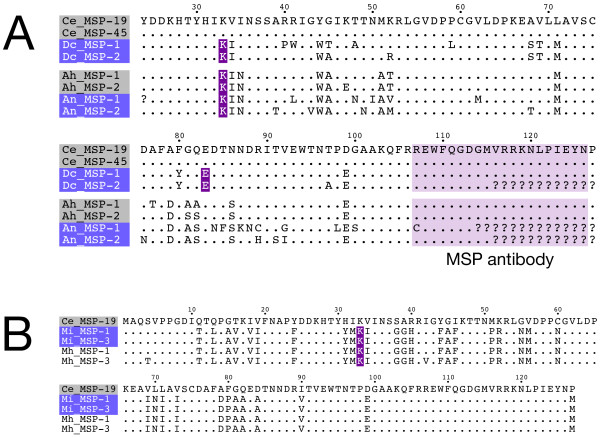
**Intact MSP genes in parthenogenetic nematodes**. **A: **Sequence alignment of deduced MSP reading frames PCR-amplified from genomic DNA of parthenogenetic nematodes. Two closely related species pairs, *D. coronatus*/*C. elegans *and *A. nanus*/*Acrobeloides (herma)*, with two representative sequences each are shown in separate alignment blocks. The position of introns is indicated (red: intron follows after this amino acid in genomic sequence), conserved AA are represented by a dot. The coding sequences of parthenogenetic species (violet) are intact, and their intron position is conserved between several species (except *C. elegans *where MSP introns are absent). The C-terminal epitope recognised by the MSP antibody is highlighted. Accession numbers: Ce_MSP-19 (AAC26926.1), Ce_MSP-45 (AAA96204.1). Amino acid numbering after Ce_MSP-19. **B: **Sequence alignment of two representative full length MSP coding regions identified in the genome sequences of the obligately parthenogenetic species *M. incognita *(violet) and the facultatively parthenogenetic species *M. hapla*. Ce_MSP-19 is included as a reference. With two exceptions (S4T, I42V), all deduced amino acid sequences in *M. incognita *and *M. hapla *are identical and highly similar to the *C. elegans *reference MSP-19. Amino acid numbering after Ce_MSP-19. The position of introns is indicated (red) and identical to the positions of other MSP proteins as shown in Figure 5A.

Figure 5A shows an alignment of representative MSP coding sequences from the two parthenogenetic nematodes and closely related hermaphroditic species. It illustrates a strong conservation of the MSP coding sequence in parthenogenetic animals (> 90% similarity to their hermaphroditic relatives). The MSP genes from parthenogenetic species were found to be intact within the range of the primers used for amplification (AA 23-114 of 127 AA full length MSP). However, part of the C-terminus relevant for binding of the MSP antibody is missing in these sequences. Therefore, we cloned the complete 3'ends of *D. coronatus *and *Acrobeloides (herma) *MSP. As in many other MSPs (Figure 4D), this region is strictly conserved in *D. coronatus *and *Acrobeloides (herma) *(Figure 5A), excluding the possibility that parthenogenetic species possess a modification within this epitope that prevents detection.

Besides intact genes with no signs of degeneration we also detected an exception in *A. nanus *where several stops in the coding region indicate a pseudogene (not shown). Pseudogenes containing frameshifts or short deletions were also present in some MSP clones from *Acrobeloides (herma) *(not shown), in agreement with a number of pseudogenes in the large *C. elegans *MSP gene family [52,53].

MSP sequences obtained from several nematode species contain introns [23,54-57]. In agreement with these reports, we predict the presence of introns in the parthenogenetic MSP sequences we cloned (Figure 5A). While intron phase is variable in previously published MSP sequences and our own, intron position and number is conserved. All introns follow the GT-AG rule [58]. In most species, including the parthenogenetic *A. nanus *and *D. coronatus*, a single intron of 60-70 bp is situated at amino acid position 33 [54-56]. *D. coronatus *is the only nematode so far where a second MSP intron is present (194 bp, after AA 83; Figure 5A). A strict conservation of intron position like in the nematode MSPs is observed in a substantial fraction of eukaryotic genes over distant taxa and may reflect an evolutionary conservation of ancient introns [59].

Our finding of apparently intact MSP genes in *D. coronatus *and *A. nanus *is based on fragments that lack the 5' and/or the 3' end of the coding regions. Therefore, we cannot exclude that mutations are present in the missing parts (22 AA and 12 AA, respectively). As whole genome sequences do not exist for either nematode, we looked for MSP coding regions in parthenogenetic species with a sequenced genome [60,61]. Via BLAST searches, we identified among partial sequences and pseudogenes a set of complete MSP coding sequences in the genomes of the obligately parthenogenetic [62] nematode *Meloidogyne incognita *and its facultatively parthenogenetic [63] sister species *Meloidogyne hapla*. We compared this MSP set to our above obtained sequences and to published MSP sequences from other, non-parthenogenetic nematodes. The deduced protein sequences of *M. incognita *and *M. hapla *are almost identical with each other (> 98%; Figure 5B). They have 73% and 67% identity to MSP sequences from *C. elegans *or *A. nanus*, respectively. Intron position (lysine 33) and number (one intron) are identical in all *Meloidogyne *MSPs investigated here and agree with our data from the parthenogenetic nematodes *A. nanus *and *D. coronatus *as well as with results from other species [54-56]. Intron length is very similar in all *Meloidogyne *MSPs (60-70 bp) and comparable to MSP sequences from other nematodes (data not shown; [55,57]). In support of our findings, a former study reported the absence of an increased evolutionary rate and of a change in the substitution pattern in MSP sequences from parthenogenetic *Meloidogyne *species [64].

To assess the translation potential of *Meloidogyne *MSPs, we investigated the regions flanking the AUG initiator codon. According to Kozak's rules, a consensus sequence surrounding the AUG codon is critical for a favourable context of translation initiation [65-67]. In all *Meloidogyne *MSP loci analysed, we find purines (A) at the -3 position and the nucleotide G in position +4 (not shown) as it was previously reported for MSP transcripts isolated from *G. rostochiensis *and *D. viviparus *[23,55]. This indicates that the respective MSP start codons fulfill the requirements for a successful initiation of translation regardless of the reproductive mode.

Thus, a remarkable conservation in genomic organisation and deduced protein sequence exists in MSPs from several parthenogenetic nematodes positioned in different clades.

## Discussion

MAP kinase activation via a hormone-dependent pathway is an essential step during oocyte-to-embryo transition of all animals. In the model organism *C. elegans*, the hormonal trigger for oocyte maturation and MAP kinase activation is MSP, a cytoskeletal protein secreted from sperm [28]. In addition, recent evidence suggests that MSP action on somatic gonadal sheath cells regulates the production, growth and meiotic maturation of oocytes [31,68]. Oocyte meiotic progression and ovulation therefore strictly depend on the presence of sperm in *C. elegans*. As different modes of reproduction exist in the phylum Nematoda, including many examples of parthenogenesis (for review see [32,33]), we investigated two key elements of meiotic progression, MAP kinase activation and the expression of MSP, in parthenogenetic nematode species that lack sperm.

### MAP kinase activation as a common theme in oocyte maturation

We have cloned MAP kinase genes from parthenogenetic nematodes. Phylogenetic analysis confirmed that they belong to the Erk1/2 subfamily of MAP kinases involved in oocyte maturation in many animals (Figure 1B). If MAP kinases of parthenogenetic nematodes had specific alterations, we would expect them to form a cluster separate from the MAP kinases of hermaphroditic species. Our analysis revealed, however, that this is not the case. The tree based on MAP kinase sequences reflects the accepted phylogenetic relationships (Figure 1B). Moreover, an 11 AA epitope which is recognised by the MAP kinase antibody is identical in Erk1/2 of humans, *C. elegans *and parthenogenetic nematodes (Figure 1C). Therefore, the MAP kinase activation we detect in parthenogenetic worms (Figure 2) is specific for Erk1/2 and corresponds to the oocyte-to-embryo transition in *C. elegans *and other animals.

Although a direct involvement of MAP kinase activation in oocyte maturation is demonstrated in *C. elegans *[34] and other systems [69,70], our data do not prove a similar role in parthenogenetic nematodes. However, considering the widespread utilisation of MAP kinase signaling for this developmental step, an alternative mechanism for meiotic maturation and cell cycle progression and hence activation of MAP kinase independently of meiotic maturation seems unlikely. Functional studies could show whether MAP kinase activation is directly required for oocyte-to-embryo transition in parthenogenetic species. Methodological limitations, however, prevent a reliable RNAi-mediated knockdown in these nematodes at the present time (PH, unpublished data). Thus, based on the presence of Erk1/2 MAP kinase activation in parthenogenetic nematodes at the time of oocyte maturation, we argue that this critical step is conserved independent of the reproductive mode.

### An elusive role for MSP in parthenogenetic nematodes

Detection of MAP kinase activity in maturing oocytes of parthenogenetic nematodes let us explore the role of MSP as a potential trigger for this activation. Using an antibody directed against the C-terminus of *C. elegans *MSP [49], we analysed MSP expression in parthenogenetic nematodes by two independent methods, immunofluorescence and Western blotting. The well-known identity of MSP C-termini in a wide range of nematodes, at least from *Ascaris *to *Caenorhabditis *(Figure 4D; [28]), already suggests a cross-reactivity of the antibody with MSPs from the *Acrobeloides *and *Diploscapter *group. Indeed, both methods were able to detect heterologous MSP of the non-parthenogenetic nematodes *Acrobeloides (herma) *and *Acrobeloides (bisex) *which are closely related to the parthenogenetic *A. nanus *(Figure 3C, E; 4A, B). Moreover, our experimental setup was sensitive enough to detect small puncta of extracellular MSP in the gonads of *C. elegans *(Figure 3A, inset) as reported in a previous paper [49]. Despite these controls, we were not able to identify even weak MSP signals in the parthenogenetic species *D. coronatus *and *A. nanus *(Figure 3G, I; Figure 4A, B). To rule out possible alterations of the antibody recognition site in parthenogenetic nematodes, we cloned MSP C-termini from the parthenogenetic *D. coronatus *and from *Acrobeloides (herma)*. We chose *Acrobeloides (herma)*, the hermaphroditic sister species to *A. nanus*, because cloning of *A. nanus *MSP was not successful. The C-terminal MSP sequences we isolated were identical to the respective *C. elegans *epitope at the amino acid level (Figure 5A), making antibody failure an unlikely explanation for the absence of a signal. Therefore, our observations are consistent with the absence of MSP at the protein level in the parthenogenetic species and with the fact that MSPs are expressed exclusively in developing spermatocytes or males in *C. elegans *and other nematodes [19-24].

In contrast to our findings, a previous paper reported the presence of MSP in two parthenogenetic species of the cephalobid group (clade 11), *Acrobeloides maximus *and *Zeldia punctata *[49]. However, evidence for MSP expression delivered in this paper is uncertain as no appropriate controls are presented. First, immunostaining of the *A. maximus *gonad detected puncta that could represent secreted MSP. However, they could also be an artefact, the more as the signal is positioned in the distal gonad where it is absent in *C. elegans *[49]. Close inspection of the data further indicates a high amount of puncta filling the entire space between oocyte nuclei. This observation would imply abundant expression of MSP in *A. maximus *and other parthenogenetic nematodes which is not the case according to our data (Figure 4).

Second, a Western blot suggests expression of MSP in the parthenogenetic species *A. maximus *and *Z. punctata*. Although we have used a different batch of the same antibody, our inability to reproduce this result can not be caused by a lower sensitivity. Expression levels of MSP in the parthenogenetic species as presented by [49] are two- to threefold weaker than in *C. elegans *and still suggest abundant expression. Using serial dilutions we showed that it is possible for us to detect at least 1% of MSP input (Figure 4C). This dynamic range demonstrates that our assay is definitely sensitive enough to detect the somewhat lower MSP expression in parthenogenetic nematodes shown by [49]. A possible reason for these discrepancies could be species-specific differences in MSP expression as we investigated different parthenogenetic *Acrobeloides *species (*A. nanus *vs. *A. maximus*). To test this assumption, we analysed cell extracts from *A. maximus *and *Z. punctata*, but MSP was undetectable in these nematodes either (Figure 4B). Thus, we were unable to reproduce MSP expression in four different parthenogenetic species.

Despite their absence as protein, we showed for the first time that intact MSP genes with features of active genes are present in parthenogenetic nematodes from several clades. Three factors may contribute - possibly in combination - to the maintenance of MSP genes in parthenogenetic nematodes:

(i) Although we could never find males in *A. nanus *and *D. coronatus*, anecdotal evidence suggests their appearance in some parthenogenetic cultures from time to time (e. g. in *A. maximus *or *Plectus mekong*; P. De Ley, personal communication; ES, unpublished; [32]). The high percentage of male progeny observed in such cultures suggests that they are able to reproduce, requiring functional sperm. Thus, MSP genes may be retained to permit occasional emergence of males in parthenogenetic species. Support for this view comes from the parthenogenetic nematode *Aphelenchus avenae *where production of numerous males upon heat or chemical treatment has been described [71,72]. Males have also been observed in the root knot nematode *M. incognita *[73,74] which is considered to be obligately parthenogenetic [62]. Although it is not known whether these examples are exceptions or the rule, they illustrate a high degree of intraspecific variation and a lack of understanding the underlying mechanisms. Obviously, a strategy of mixing parthenogenesis with sexual reproduction instead of pursuing parthenogenesis alone can be evolutionarily beneficial.

(ii) The preservation of MSP genes in parthenogenetic nematodes may be explained by a recent transition to parthenogenesis. Evolution did not yet modify MSP genes, and although they are obsolete, we find them still intact. A model where parthenogenesis constantly emerges and disappears in different branches of nematodes could account for these observations. A feature of this model, supported by theoretical considerations [75,76], is that parthenogenesis is disadvantageous and destined for extinction on the long run. Following this idea, parthenogenesis can be observed in different branches of the nematode tree at a given time, but it is always of recent origin because disadvantages prevent its long-term preservation.

Although this is an attractive hypothesis, presently no data exist for its direct proof which would e. g. require the inactivation of intact MSPs in a parthenogenetic background. Nevertheless, a detailed comparison of MSP sequences in non-parthenogenetic vs. parthenogenetic root knot nematodes proposed a recent origin of parthenogenesis based on the absence of an increased substitution rate, changed substitution pattern or pseudogene formation [64]. These results, however, could also be explained assuming a strong selective pressure on the maintenance of MSP genes, as in scenario (i) and (iii).

(iii) As a third possibility, MSP genes might be conserved because MSP is needed to trigger oocyte maturation in parthenogenetic species like in *C. elegans*. Albeit this view is not supported by our data, we cannot exclude the presence of MSP at levels too low for detection via Western blotting and immunofluorescence. MSP is deprived of structural and motility functions in sperm-less parthenogenotes. Thus, a low expression level could be sufficient for acting like a hormone in MAP kinase signaling and oocyte maturation. This model would implicate a shift in MSP expression from sperm to non-sperm cells which could be achieved by mutations in regulatory sites. As *C. elegans *MSP is exported from sperm by a non-classical vesicle budding mechanism [49], one would further expect a similar mechanism in oocytes or sheath cells of parthenogenetic nematodes which is not known at the present time. Despite the absence of direct experimental support, this last interpretation would require only minimal changes in the initial hermaphroditic system. Existing signaling pathways could be used to control oocyte maturation after transition to parthenogenesis.

With the presently available data it is not possible to distinguish between the presented alternatives. Unlike the first two scenarios, the last hypothesis provides a number of reasonable predictions and may serve as a starting point for further analysis.

## Conclusions

The *C. elegans *oocyte-to-embryo transition is governed by MSP, a cytoskeletal protein released from sperm, and is therefore coupled to the presence of sperm. Within the Nematoda, reproduction is highly versatile, and parthenogenesis, reproduction without the contribution of sperm, has evolved many times independently. To elucidate oocyte-to-embryo transition in nematodes that lack sperm, we have investigated two of its hallmarks, MAP kinase activation and MSP signaling, in two representative parthenogenetic species. Activation of MAP kinase in parthenogenetic nematodes at the time of oocyte maturation implies that this signal does not constitute a simple switch whose presence or absence determines the reproductive mode. Instead, it might regulate meiotic cell cycle progression of maturing oocytes independent of the reproductive mode.

MSP, the major sperm protein, has no apparent function in parthenogenetic nematodes that lack sperm. Indeed, we demonstrate its absence at the protein level. However, the unexpected discovery of intact MSP genes suggests either their strong maintenance by natural selection or a very recent origin of parthenogenesis in the nematode branches we examined.

## Methods

### Nematode cultivation and strains

All nematodes were cultivated under standard conditions [77], feeding on agar plates with *E. coli *OP50 as a food source. To reduce contamination with other bacteria, we used minimal medium plates [78]. Studies were carried out with the following strains: *C. elegans *N2 (hermaphroditic [77]), *Acrobeloides sp*. PS1146 (hermaphroditic; kindly provided by Marie-Anne Felix, France; for simplicity called *Acrobeloides (herma)*), *Acrobeloides sp*. 0/5061 (gonochoristic; kindly provided by Walter Sudhaus, Berlin, Germany; for simplicity called *Acrobeloides (bisex)*), *Acrobeloides nanus *ES501 (parthenogenetic [79]; called *A. nanus*), *Diploscapter coronatus *PDL0010 (parthenogenetic; kindly provided by Paul De Ley, University of California, Riverside; called *D. coronatus*).

### Immunostaining and Western blot

For visualisation of MAP kinase and MSP, we prepared and immunostained nematode gonads as described [28,80]. Briefly, ≥ 50 nematodes were dissected in 1 × Egg Salts containing 0.01% Levamisole (Sigma). After fixation in 3% Formaldehyde (Science Services) for 1 h, specimens were washed (PBST) and incubated either with a monoclonal antibody against MAP kinase (1:1.500) that only recognises the activated, diphosphorylated form of the Erk1/2 activation loop (Sigma # M9692) or with a monoclonal antibody (1:400) raised against the C-terminus of *C. elegans *MSP [49]. Secondary antibody was an Alexa Fluor^® ^488 coupled goat anti-mouse antibody at a 1:1.000 dilution (Invitrogen). Microscopy was carried out with a Zeiss Axioskop 2 fluorescence microscope. Pictures were taken with an AxioCam MRc camera (Zeiss) and arranged with the Keynote software (Apple).

For detection of MSP in nematode lysates, 40 mg animals (mixed stage population) of the respective species were washed in H_2_O, freeze-cracked twice in liquid nitrogen/ice, and boiled for 10 min in SDS loading buffer. After electrophoresis on a 12% SDS gel, proteins were electroblotted onto PVDF membranes under semidry conditions and subjected to Western detection. Primary antibody was the monoclonal anti-MSP antibody directed against the C-terminus of MSP [49] at 1:400 or, as a control, a 1:3.000 dilution of a mouse monoclonal anti-*α*-tubulin antibody (Sigma # T9026). Secondary antibody was a HRP-coupled goat anti-mouse antibody at 1:2.500 (Santa Cruz Biotechnology, Inc.). Antibodies were diluted in TBST/2% milk powder. Autoradiographic films (Fuji) were developed according to standard protocols, digitally scanned and processed with Keynote.

### Cloning of MAP kinase and MSP genes

To clone MAP kinase sequences, RNA was extracted from various nematodes and cDNA was synthesised as described earlier [81]. Initially, the conserved kinase regions of MAP kinase were PCR amplified from cDNA using degenerate primers. The corresponding 5'ends were obtained via PCR with a gene specific reverse primer and the conserved SL1 splice leader [82] as forward primer. The 3'ends were fished with a gene specific forward primer and the reverse primer mixture of the Smart RACE protocol (Clontech Laboratories, Inc.). For cloning of the MSP reading frames, genomic DNA of the respective nematodes was extracted with standard methods [83]. A combined degenerate and inverse PCR approach was employed to obtain genomic MSP fragments that included the 3'end. Amplification products were cloned into the pJet1 vector (Fermentas), transformed into *E. coli *XL1-Blue bacteria (Stratagene) and sequenced with the BigDye^® ^Terminator V3.1 sequencing kit (Applied Biosystems). Sequence assembly and editing were carried out using the Phred/Phrap/Consed package [84,85] and the plasmid editor ApE http://www.biology.utah.edu/jorgensen/wayned/ape/. The mentioned MAP kinase and MSP sequences were deposited at the EMBL Nucleotide Sequence Database (accession numbers: FN667822-FN667824 for MAP kinase, FN433114-FN433119 for MSP sequences).

### Detection of MSP genes in Meloidogyne

Whole genome sequences from *M. incognita *and *M. hapla *[60,61] were downloaded from NCBI and translated to the six open reading frames using Emboss [86]. After constructing BLAST databases [87], BLASTP searches identified MSP coding contigs within the respective genomes. Based on this information, MSP genes were annotated using Artemis [88] and the resulting sequences were deposited at the EMBL Nucleotide Sequence Database (accession numbers: FN433120-FN433123). Methods for sequence alignment and presentation see below.

### Sequence alignment and phylogenetic analysis

For alignment and phylogeny of MAP kinase proteins, following MAP kinase sequences were retrieved from NCBI http://www.ncbi.nlm.nih.gov/protein: *C. elegans *MPK-1 and MPK-2 (Accession numbers P39745.2 and AAN60533.1), human and zebrafish Erk1 (P27361.4 and AAY57804.1), human Erk2 (NP_620407.1), human and zebrafish Erk5 (Q13164.2 and ABC94477.1), human, zebrafish and *C. elegans *p38 (Q16539.3, NP_571797.1 and AAM98016.1), human and zebrafish Jnk (NP_620707.1 and NP_571796.1), zebrafish Erk3 (ABC94479.1) and human Erk4 (NP_002738.2). A protein multiple sequence alignment was computed from the retrieved and our isolated MAP kinase sequences using Muscle [89]. The alignment was trimmed to the well alignable region of roughly the serine-threonine kinase domain (317 amino acid positions) in SeaView [90]. Details of aligned regions were produced using TEXshade [91]. Phylogenetic trees of the MAP kinase dataset were computed using the maximum likelihood method. First, the optimal model of sequence evolution was determined by ProtTest version 2.0 [92] according to the Akaike Information Criterion. The resulting optimal model (RtREV) was used for maximum likelihood with the program PhyML version 3.0 and 100 bootstrap replicates [93]. The initial tree was visualised with Phylip [94] and edited with Adobe Illustrator software.

## Authors' contributions

PH performed most experiments and wrote the paper. MK and NN cloned the MAP kinase genes. NN and PH cloned the MSP genes. MK, ES, and PH designed the study and discussed the results. ES critically revised the manuscript. All authors read and approved the final manuscript.
